# Basaltic Terrains in Idaho and Hawai‘i as Planetary Analogs for Mars Geology and Astrobiology

**DOI:** 10.1089/ast.2018.1847

**Published:** 2019-03-06

**Authors:** Scott S. Hughes, Christopher W. Haberle, Shannon E. Kobs Nawotniak, Alexander Sehlke, W. Brent Garry, Richard C. Elphic, Samuel J. Payler, Adam H. Stevens, Charles S. Cockell, Allyson L. Brady, Jennifer L. Heldmann, Darlene S.S. Lim

**Affiliations:** ^1^ Department of Geosciences, Idaho State University, Pocatello, Idaho.; ^2^ Mars Space Flight Facility, School of Earth and Space Exploration, Arizona State University, Tempe, Arizona.; ^3^ NASA Ames Research Center, Moffett Field, California.; ^4^ NASA Goddard Space Flight Center, Greenbelt, Maryland.; ^5^ UK Centre for Astrobiology, School of Physics and Astronomy, University of Edinburgh, Edinburgh, United Kingdom.; ^6^ School of Geography and Earth Sciences, McMaster University, Hamilton, Ontario, Canada.; ^7^ NASA Headquarters, Washington, District of Columbia.; ^8^ BAER Institute, Moffett Field, California.

**Keywords:** Volcanic terrains, Planetary analogs, Field regions, Basalt, Rock alteration

## Abstract

Field research target regions within two basaltic geologic provinces are described as Earth analogs to Mars. Regions within the eastern Snake River Plain of Idaho and the Big Island of Hawai‘i, the United States, provinces that represent analogs of present-day and early Mars, respectively, were evaluated on the basis of geologic settings, rock lithology and geochemistry, rock alteration, and climate. Each of these factors provides rationale for the selection of specific targets for field research in five analog target regions: (1) Big Craters and (2) Highway lava flows at Craters of the Moon National Monument and Preserve, Idaho, and (3) Mauna Ulu low shield, (4) Kīlauea Iki lava lake, and (5) Kīlauea caldera in the Kīlauea Volcano summit region and the East Rift Zone of Hawai‘i. Our evaluation of compositional and textural attributes, as well as the effects of syn- and posteruptive rock alteration, shows that basaltic terrains in Idaho and Hawai‘i provide a way to characterize the geology and major geologic substrates that host biological activity of relevance to Mars exploration. This work provides the foundation to better understand the scientific questions related to the habitability of basaltic terrains, the rationale behind selecting analog field targets, and their applicability as analogs to Mars.

## 1. Introduction

NASA's BASALT (Biologic Analog Science Associated with Lava Terrains) research program comprises an international team of scientists, engineers, mission operators, and astronauts investigating martian habitability and the technologies necessary for human/robotic exploration of Mars. The driving goal of the BASALT program is to integrate three Mars-focused disciplines (science, operations, and technology) to inform future human spaceflight activities (*e.g*., Lim *et al.*, 2019; Payler *et al.*, 2019). During field campaigns, the methods within each discipline are designed to seamlessly provide a high-fidelity simulation of human spaceflight concepts of operation for science-driven exploration of the martian surface. Accordingly, the BASALT research program enables the connection between what can be learned before exploration to knowledge that becomes available during a ground-based mission. We focus on Earth analog regions that will provide insight into the scientific questions related to habitability of specific types of basaltic terrains. This article presents an overview of the rationale behind the BASALT analog field targets and their applicability as planetary comparisons to Mars during relatively early and late periods of Mars' geologic history.

The ability to identify evidence of habitable environments on Mars is a high priority within the scientific community and the Mars Exploration Program Analysis Group (MEPAG, 2015). BASALT aims to help define what constitutes a habitable Mars environment in terms of geologic substrate and climate, and to address both biological and geological questions related to the search for extant and extinct life on Mars. The BASALT program is driven by the hypothesis that the geologic substrate will affect the diversity and biomass of life, which will vary with different combinations of rock composition, texture, and alteration condition. To test the correlation between geology and biologic activity and to evaluate possible Mars-like scenarios, geologic analogues and the biota within and on solid rock are closely examined for potential associations and how the microbiota interact with the rock itself. Results are expected to provide clues to the habitability and potential types of life that could have proliferated early in Mars' history, or may yet still be active.

**FIG. 1.**
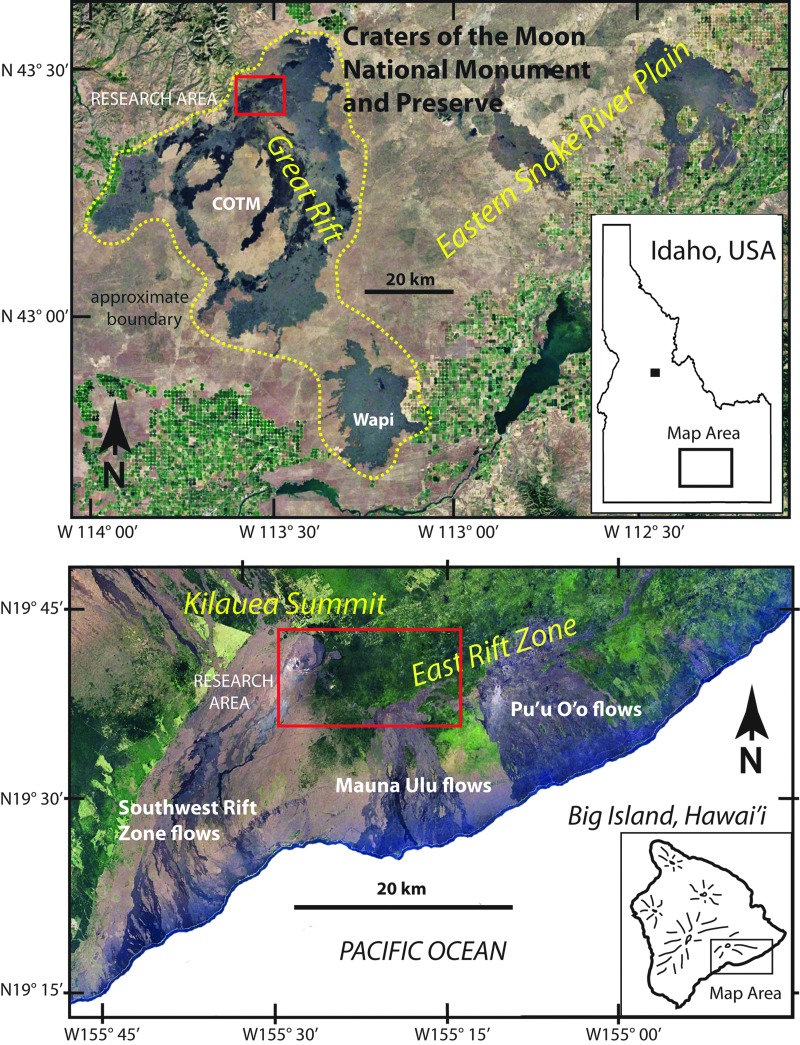
Landsat images (Google Earth^®^, Copernicus imagery) in the vicinity of research areas (red boxes) at COTM on the eastern Snake River Plain, Idaho, (upper) and Kīlauea Volcano on the Big Island of Hawaii (lower). COTM, Craters of the Moon National Monument and Preserve.

Analog field areas in Idaho and Hawaii, the United States (Fig. 1), and the regions of interest (ROIs) within, were chosen based on their heritage as planetary analogues (*e.g*., Greeley, 1974, 1982; Greeley and King, 1977) and for volcanic processes known to have occurred during Mars' history (Carr, 1973, 2006; Carr and Head, 2010), which attest to their potential to serve as appropriate astrobiology analogues. At our field targets, as on Mars, the composition of material available for physical and chemical provision of nutrients and energy for biologic activity is predominantly basaltic lava and related lava types such as hawaiite and latite. In particular, we address the various types of rock alteration, including characteristic minerals, textures, local settings, and scales, influencing the availability of nutrients and energy sources for biota within these chosen analog regions. We discuss their geologic attributes in terms of two primary goals: (1) to assess the compositional and textural diversity that characterizes the ROIs and (2) to apply these attributes and the information gained from field experiences (and subsequent laboratory work) to Mars geology and what might be observed at comparable sites on Mars.

## 2. The Influence of Rock Alteration

Diversity in volcanic rock types (chemistry, mineralogy, texture) may be derived through either magmatic evolution or processes related to the subsequent alteration of pristine rock. Primary magmatic processes, related to volcanism style and geologic setting, lead to differentiated products that may comprise a wide spectrum of compositions in any volcanic province. Subsequent changes to rock mineralogy and texture via alteration can dramatically affect the availability of CHNOPS elements and other chemical species used by biology (Uroz *et al.*, 2009). Responses to alteration conditions include devitrification, oxidation, dissolution (with potential loss of mobile elements), secondary mineral deposition, and textural modifications related to these transformations. Although we know that basaltic terrains can host a rich diversity of microbial life (*e.g*., Dunfield and King, 2004; Costello *et al.*, 2009; Kelly *et al.*, 2011; Cockell *et al.*, 2011a, 2011b, 2013), we still have very little understanding of how the geological environment influences microbial community structure and habitability. Determining the most appropriate locations for sample acquisition is therefore directly related to the condition of rock alteration, which can greatly affect texture, lithology, and chemistry.

### 2.1. Types of rock alteration

Processes related to rock alteration include the oxidizing effects of high-temperature, syn-eruptive volcanic gases, secondary fumarolic or other hydrothermal reactions, low-temperature weathering by meteoric fluids, and physical weathering and reworking. All of these can contribute to a host of variant compositions and textures. For the purpose of this study, mineral assemblages fall into two broad categories: primary and secondary. Primary minerals are those that crystallize from magma (pre- or syn-eruptive conditions), while secondary minerals are formed through the subsolidus alteration of primary minerals by the interactions of aqueous fluids, gases, or heat.

Basalt is a fine-grained, mostly crystalline igneous rock composed of varying amounts of plagioclase feldspar and pyroxene often forming with other components, including olivine, oxides, glass, and minor accessory minerals such as apatite and sulfides. These primary constituents are all nominally anhydrous. Following the emplacement of a fresh lava flow, a variety of secondary processes can act to form secondary minerals and textural modifications that will reflect the alteration process. The extent of alteration depends on the temperature, length of exposure time, and the compositions of the primary rock substrate and fluids/gases involved. Alteration conditions can be relatively hot and dry (syn-eruptive), hot and wet (hydrothermal or fumarolic), cool and wet (long-term meteoric water interaction), or even cool and dry (high desert conditions). Wet conditions can entail additional complexities related to relatively high or low pH and the concentration of dissolved solids.

**FIG. 2.**
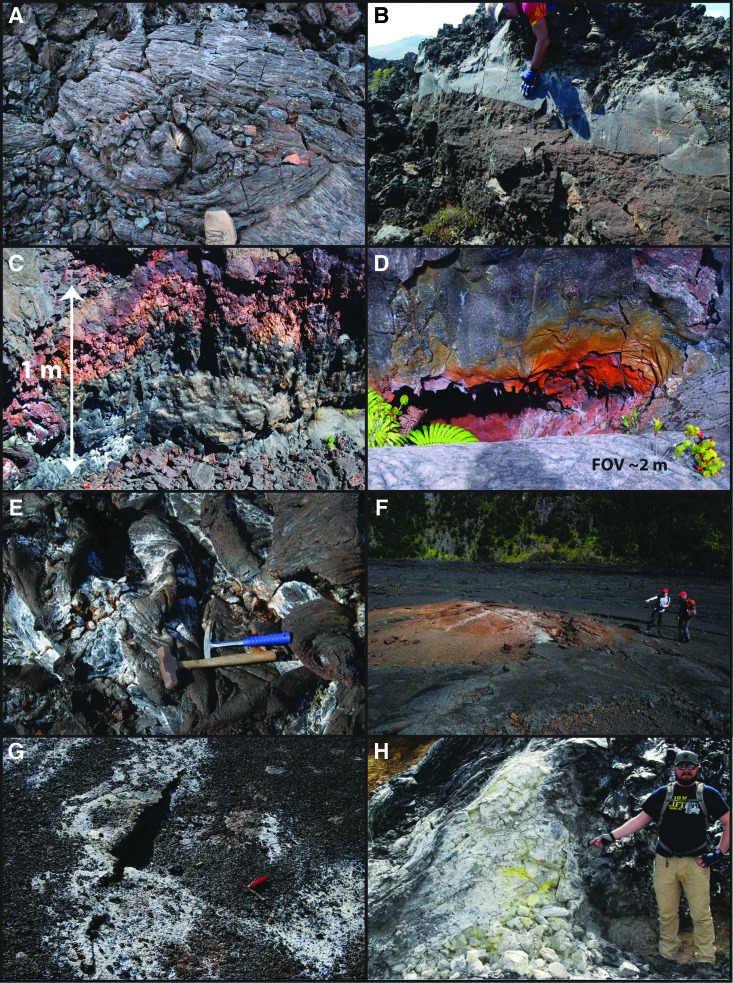
Examples of rock textures and alteration exhibited in Idaho and Hawaii. **(A)** Minimal syn-emplacement alteration (slight reddish oxidation) of Big Craters flow surface, **(B)** dense and frothy primary textures in Highway flow, **(C)** high-temperature oxidation in Mauna Ulu lava, **(D)** high-temperature oxidation cavity on Mauna Ulu shield, **(E)** relict meteoric fumarole on Mauna Ulu flank lavas, **(F)** active meteoric fumarole on Kīlauea Iki lava lake, **(G)** relict fumarole on Kīlauea Iki lava lake, **(H)** recently active magmatic fumarole with native sulfur deposits on margin of Kīlauea caldera.

Our sampling strategy was intended to provide significant information on these secondary alteration processes relevant to their biological habitability. Variations in alteration features found in cracks, gas cavities, pits, mounds, channels, and so on were highly relevant to the selection of targeted sampling locations, so each class of alteration is considered individually in the following sections. While field visual inspection and *in situ* analyses (*e.g*., Sehlke *et al.*, 2019) help to identify the most attractive sample locations during field work, confirmation typically must rely on subsequent laboratory analyses of replicates (*e.g*., Cockell *et al.*, 2019). Whether assessed during field work or in laboratory experiments, the types of alteration important to this program, in terms of temperature, process, and implied exposure times, are summarized as follows (Fig. 2).

#### 2.1.1. Unaltered rock

Unaltered rock is generally outcrop (flow lobe, spatter rampart, lava surface) material consisting of basalt that has not been altered by volatiles. Rock is typically black/dark gray with dense to vesicular textures. Outcrops with minimal alteration may be considered essentially “unaltered” with respect to secondary mineralization, especially in locations where significant textural variability is related to syn-eruptive processes (Fig. 2A, B). It is expected that unaltered material will be located away from vents, cracks, and active fumaroles. Unaltered outcrops may also be found where the rock has been protected (by overburden, dry climate, etc.) from long-term exposure to weathering agents.

#### 2.1.2. Syn-emplacement alteration

Alteration is caused by the high-temperature, near-instantaneous, oxidizing effect of volcanic gases derived during an eruption (Fig. 2C, D). Fresh lava and tephra, normally appearing vitreous steel-gray to black at the time of eruption, becomes intensely red due to the oxidation of iron (from ferrous to ferric redox species), which is concomitantly associated with other changes such as devitrification of interstitial glass. The hot gases that cause alteration are released directly from the lava itself, or less likely, from its interaction with underlying moist ground. Syn-eruptive alteration typically results in patches of bright colors, including red, orange, yellow, and even purple.

#### 2.1.3. Alteration by fumaroles

Alteration of rock is caused by steam and/or magmatic gases (H_2_O, CO_2_, SO_2_, HCl, HS, and F) released following emplacement or development of the volcanic feature (lava flow, cone, fissure, crater, etc.). Magmatic gases tend to have relatively low pH due to the addition of carbonic and sulfur-containing components to aqueous fluids (*e.g*., H_2_O + SO_2_ = H_2_SO_4_, sulfuric acid) that result in chemical breakdown of primary minerals such as plagioclase to produce clay minerals (Minitti *et al.*, 2007; Gerard and McHenry, 2012). Fluids may be derived directly from the magma or indirectly from meteoric water. The relative proportions of meteoric to magmatic components may vary considerably in a given volcanic terrain. Steam produced by the interaction of circulating water with hot lava or fissures often leads to fumarolic activity without significant mineralization related to typically acidic volcanic gases. In some cases, these fumaroles (Fig. 2F) may actually contain magmatic components that have been leached out and become incorporated into the emitted gas. Solfatara is the name given to typically magmatic fumaroles in basaltic terrains, that is, those that produce sulfurous fumes usually derived from intrinsic volcanic gases.

In general, fumarolic mineral deposits (Fig. 2H) include common varieties of native sulfur, sulfides, sulfosalts, sulfates, halides, oxides, hydroxides, carbonates, and borates. Temperatures higher than surroundings, vent gases, and precipitating minerals all contribute to the creation of a relatively hostile environment that may be colonized by extremophiles, which is one of the most significant research topics within the astrobiology community (*e.g*., Wynn-Williams *et al.*, 2001; Pikuta *et al.*, 2007; Chang, 2016). Microbial communities will likely vary in composition with distance from a hotter interior fumarole toward more clement conditions.

Fumaroles that have gone extinct or are currently dormant may be viable as places for alteration processes to continue for months or years after gases have stopped being emitted. Inactive fumaroles also may be associated with active ones in the vicinity, a relation that is common in large fumarole fields where activity is spread out over several tens or even hundreds of meters. Alteration products in relict fumaroles (Fig. 2E, G) likely comprise secondary material introduced by fluids or directly deposited sublimates that are somewhat resistant to weathering processes.

#### 2.1.4. Ambient temperature alteration

This form of “low-temperature” alteration is essentially weathering related to the long-term effects of subaerial or subaqueous chemical change under ambient climate conditions (Dessert *et al.*, 2003; Adcock *et al.*, 2018). Meteoric water is generally important, in the form of precipitation, seepage, or atmospheric moisture, which can result in chemical breakdown of minerals and glass, and also provide for transport and deposition of chemical constituents of secondary minerals such as calcite, sulfates, smectites, and zeolites (*e.g*., Stefánsson and Gíslason, 2001; Richardson *et al.*, 2012; Mattioli *et al.*, 2016; Kanakiya *et al.*, 2017).

Chemical breakdown of primary minerals, regardless of size, by normal low-temperature weathering or hydrothermal activity depends on temperature, pH, crystal composition, and structure. The long-term resistance to weathering of dominant minerals in basaltic rocks generally decreases in the order: plagioclase–pyroxene–glass–olivine (*e.g*., Hausrath *et al.*, 2008). Olivine, the least resistant, may decompose within a few thousand years in warm, moist climates and thus may contribute to accumulation of available Fe and Mg for biologic activity. Volcanic glass may take much longer (several hundred k.a.); however, much faster rates of decomposition for all minerals are expected under nonambient conditions.

## 3. BASALT Field Target Regions

Targeted areas for field research are typically defined by scales ranging in size from planetary to outcrop. Planetary scale research areas are geologic provinces, such as the Big Island of Hawaii or the eastern Snake River Plain (ESRP) of Idaho. Designations adopted by the BASALT program for smaller size areas (Table 1) that lie within major provinces are as follows: zone, region, station, and location (in order of decreasing size). A zone is depicted as a geologic system (100s of km^2^) within a province, such as the East Rift Zone (ERZ) and Kīlauea Volcano in Hawaii or Craters of the Moon National Monument and Preserve (COTM) and the Great Rift in Idaho. Regions are set within zones and refer to specific features, generally <10 km^2^, that derive from a given event. Examples include Mauna Ulu shield volcano on the ERZ and the Highway Flow at COTM. Here we provide the geologic details of target regions. While smaller subdivisions are not considered in detail, many of the features used as rationale for target region selection occur at the scale of station (∼10–100 m diameter, tens of m^2^) or location (1 m^2^ outcrop). Specifically, we focus on regions within the volcanic terrains of COTM (Fig. 1, upper), and the Kīlauea Volcano summit region and ERZ of Hawaii Volcanoes National Park (HAVO) (Fig. 1, lower).

**Table 1.** Terms Used by BASALT to Designate Relative Scales of Field Research Areas

*Term*	*Scale*	*Examples*
Province	Hundreds of kilometers (geologic terranes)	Hawaiian Islands, Snake River Plain
Zone	Tens of kilometers	COTM, ERZ, Kīlauea Volcano
Region	1–10 km	Mauna Ulu, Kīlauea Iki, Highway Flow, North Crater Flow
Station	10 m diameter (2016, ID and HI); up to 100 m (2017 HI)	Outcrop, deposit or cluster of features having similar visible characteristics
Location	∼1 m (Outcrop selected for sample)	Collection point on outcrop
Replicate	∼1 m (Precisely where sample extracted)	Collection point on outcrop

BASALT, Biologic Analog Science Associated with Lava Terrains; COTM, Craters of the Moon National Monument and Preserve; ERZ, East Rift Zone.

### 3.1. Diversity in volcanic provinces

**FIG. 3.**
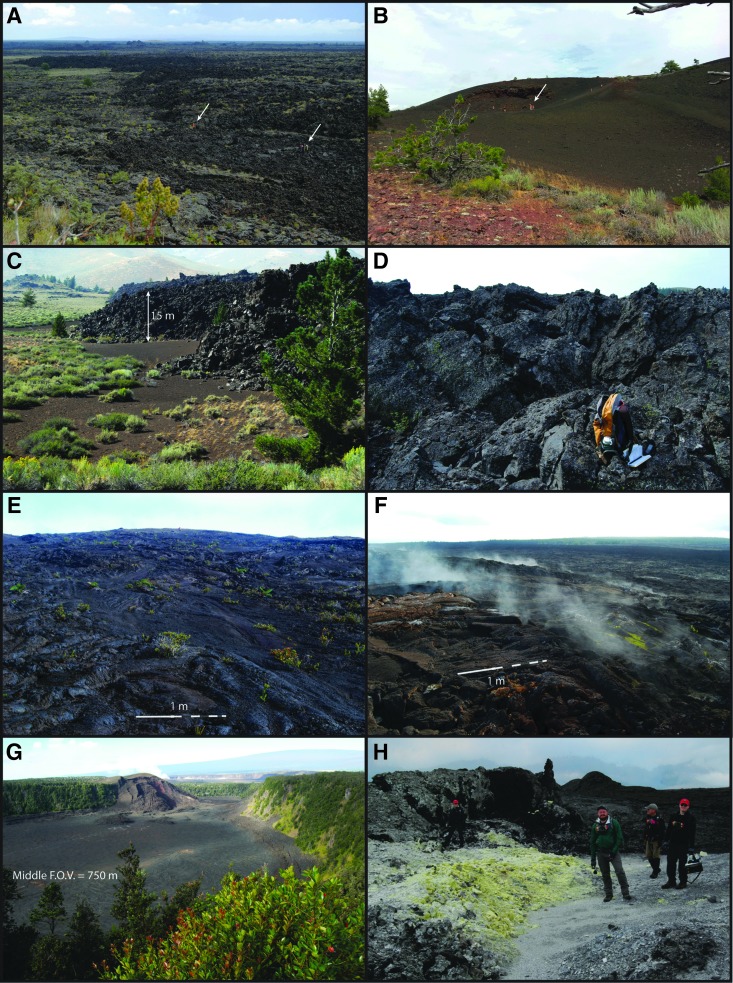
Lava flow surfaces and volcanic features of potential target regions in Idaho and Hawaii. **(A)** Big Craters lava flow, **(B)** Big Craters vent area (in foreground; white arrows point to people for scale), **(C)** Highway flow western margin, **(D)** Highway flow surface, **(E)** Mauna Ulu flank lavas, **(F)** Mauna Ulu SW flank with active (meteoric) fumaroles, **(G)** Kīlauea Iki lava lake and eruptive vent, **(H)** Solfatara fumarole deposits along fissure in Kīlauea caldera near SE margin.

Volcanic provinces in Idaho and Hawaii represent significant diversity in geologic setting and lava flow surface types. Lava flow surfaces vary across a wide spectrum of morphologies (Fig. 3A–H), exhibiting features that provide conditions for biologic activity. Aside from syn- or postemplacement chemical alteration, each type has surficial and internal characteristics related to intrinsic eruptive properties (temperature, composition, density, viscosity, etc.) and the response to external physical stresses during emplacement (magma supply rate, slope, cooling conditions, etc.). The structures that form in response to these stresses are characterized by differences in primary textures and types of alteration, including secondary mineralization.

The textures of basalt and derivative volcanic rocks are mainly microcrystalline or glassy, some with small (less than a few mm) crystals (phenocrysts) of primary minerals such as plagioclase, olivine, and pyroxene. Many groundmass textures, that is, the material between crystals, are glassy in appearance to the naked eye and even under microscopic analysis, but they actually may have a submicroscopic crystalline texture that is discernible only via electron microscopy. One example is the Big Craters (BC) lava flow (Fig. 3A) that has a rough glassy surface at cm-scale due to ripped vesicles caused by physical stretching during emplacement.

#### 3.1.1. Selection of target regions

**FIG. 4.**
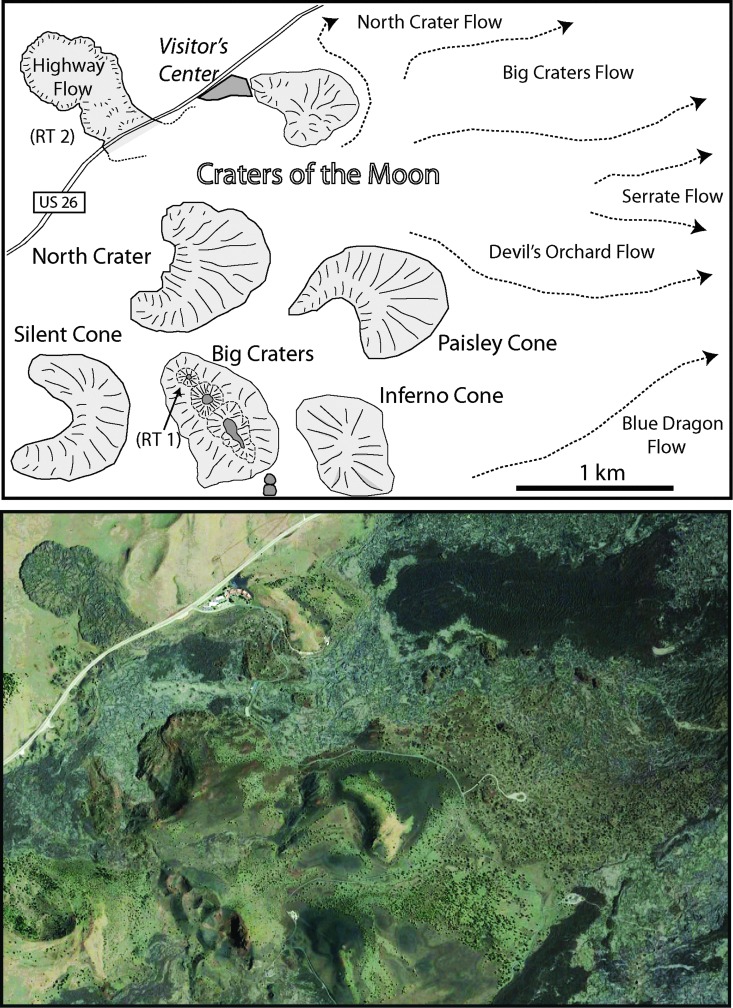
Research area (outlined in Fig. 1, upper) in the northern part of COTM. Upper: outline of major volcanic features and research target regions RT 1 (Big Craters vent) and RT 2 (Highway flow). Lower: Landsat image (Google Earth) of map area. RT, research target.

**FIG. 5.**
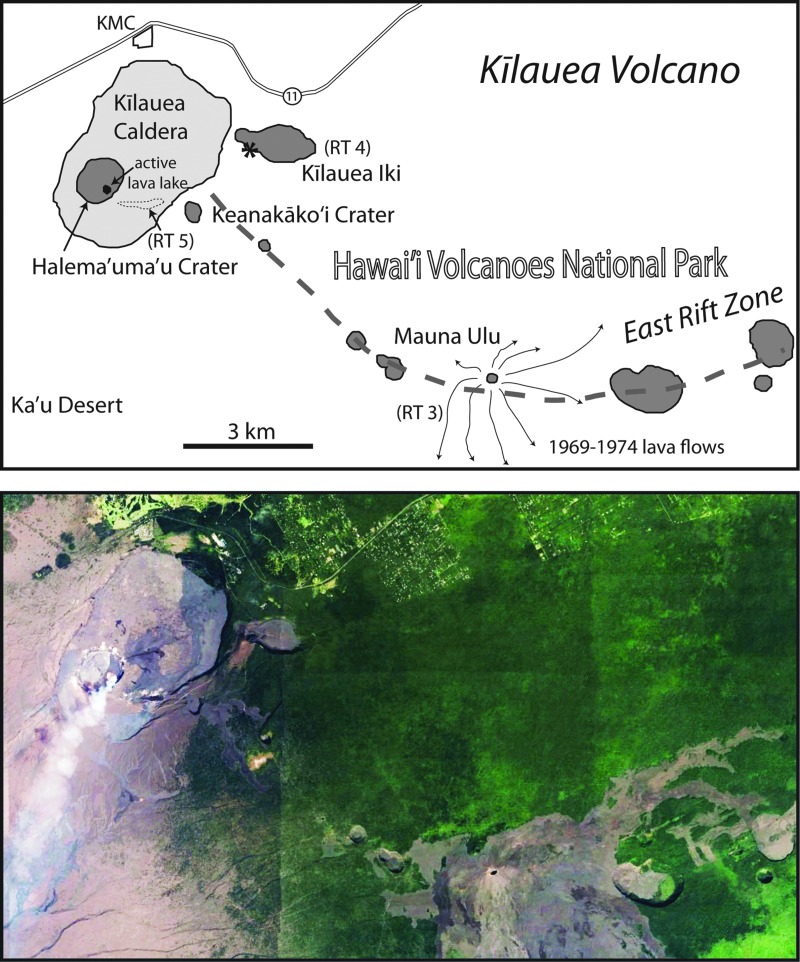
Research area (outlined in Fig. 1, lower) near Kīlauea summit and proximal part of the East Rift Zone. Upper: outline of major volcanic features and research target regions RT 3 (Mauna Ulu), RT 4 (Kīlauea Iki), and RT 5 (caldera floor SE of Halema‘uma‘u Crater). Lower: Landsat image (Google Earth) of map area.

Selection of BASALT field targets on Earth was based on the premise that the biomass and diversity of life that can proliferate on volcanic rock substrates are influenced by conditions of surface morphology, age, weathering, and rock chemistry, including chemical alteration. Research targets (RTs) in Idaho (Fig. 4), and Hawaii (Fig. 5) are similar in some respects and serve as complementary systems, with differences in geologic setting, climate, and age that can be explored as analogues to Mars. Both largely represent basaltic systems characterized by eruptive fissures, extension fractures, low shields, and cones that depict plains volcanism (Greeley, 1982), known to have occurred on Mars (*e.g*., Greeley and Spudis, 1981; Plescia, 1981, 2004; Carr, 2006; Hauber *et al.*, 2009, 2011). Geochemical diversity spans a large range from pristine basalt to evolved, more silicic and/or more alkalic compositions in both systems. Intermediate compositions, including trachybasalt (hawaiite), basaltic trachyandesite (benmoreite), and trachydacite (latite), are derived from a basaltic parent.

While the ERZ remains volcanically active in a relatively warm, moist tropical climate, the Great Rift is currently inactive in a cooler, drier climate (Table 2). Kīlauea eruptions are historical, with active fumaroles emanating from recently erupted lava flows; however, the most recent eruptions on the ESRP, including COTM, occurred a little over 2000 years ago (Kuntz *et al.*, 1992, 2007). Weather near the summit of Kīlauea and the ERZ varies daily from rainy to sunny throughout the year; whereas conditions at COTM and the northern segment of the Great Rift are characterized by hot, windy, sunny summers and cold, snowy winters.

**Table 2.** Weather Conditions in Target Regions from Online Sources

*Region*	*Elevation*	*Average maximum temperature (July)*	*Average minimum temperature (January)*	*Annual total precipitation*	*Annual snowfall*
Kīlauea (summit)	1220 m (4000′)	28°C (83°F)	18°C (64°F)	1.75 m (69″)	0
COTM (visitors Ctr.)	1800 m (5910′)	29°C (85°F)	−12°C (11°F)	0.40 m (15.5″)	2.27 m (89.4″)

*Sources:* www.nps.gov/havo/index.htm; www.nps.gov/crmo/index.htm

#### 3.1.2. Geologic settings

The Great Rift–COTM system on the ESRP and the Kīlauea–ERZ system on the Big Island of Hawaii represent, respectively, continental and oceanic intraplate volcanism (*i.e.*, not associated with active plate margins). This primary difference is the foremost rationale for selecting these zones, namely that oceanic and continental lithospheres have significantly different compositions and structures. Volcanic derivatives, therefore, will exhibit differences in compositional trends reflected in geochemistry, mineralogy, and texture. Regardless, both systems have been attributed in some degree to volcanic hotspots, generated by mantle thermal plumes from deep sources. They both exhibit deposits related to volcanic fissure eruptions along magmatic rift zones and have many similar surface constructs such as spatter ramparts and cones, pāhoehoe and ‘a'ā lava flows, and self-leveed stagnated lava ponds.

While similarities between both systems are inherent in closely matched volcanic features, fundamental differences exist in lava composition and, by inference, the processes of magma genesis. Besides the overall contrast between oceanic and continental settings, one notable factor in volcanic evolution is how each system is related to a mantle plume. The Big Island of Hawaii is an oceanic island situated on, or very near, an active (mantle plume) hotspot (*e.g*., Wilson, 1963a, 1963b; Clague and Dalrymple, 1987; Clague, 1996; Sherrod *et al.*, 2007), whereas the COTM system on the ESRP is situated in the time-transgressive track (the “wake”) of the Yellowstone hotspot that was in the vicinity ∼10 m.y. ago (*e.g*., Armstrong *et al.*, 1975; Pierce and Morgan, 1992; Smith and Braile, 1994). Thus, ESRP magma genesis may not be directly related to the Yellowstone hotspot, which left the vicinity long before recent eruptions.

Significant differences exist between the two systems in lava composition, emplacement age, and climate, all of which are important factors that can influence the bioavailability of nutrients. In terms of volcanic activity and climate, the relatively dry climate and currently inactive volcanic pile at COTM are used to represent present-day late Amazonian Mars. The moist climate and active volcanism at Kīlauea and the ERZ are intended to represent late Noachian through Hesperian and possibly early Amazonian Mars, when volcanism was more extensive early in Mars geologic history. Understandably, these representations are not exact analogues; however, the current knowledge of Mars' geologic history (*e.g*., Head *et al.*, 2006; Carr and Head, 2010; Hauber *et al.*, 2011) suggests that they are appropriate for the evaluation of similar processes that could affect biologic activity in volcanic terrains. Justification for this two-pronged approach is inherent in the notion that biologic activity may have either proliferated early in Mars' history and died out, or life is currently viable and may ultimately be discovered (or introduced) in some locales on the planet.

**FIG. 6.**
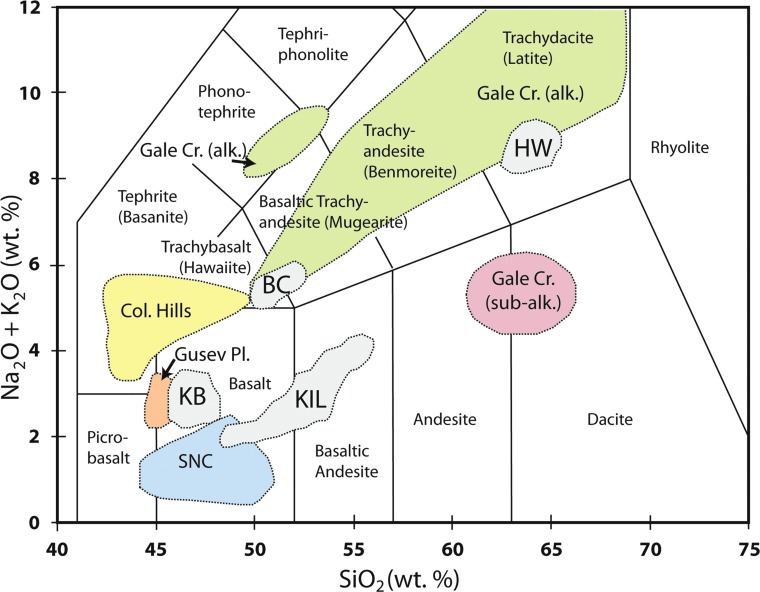
Total alkalis (Na_2_O + K_2_O) versus SiO_2_ diagram depicting outlines of compositions in study areas and compositional fields from Mars: KIL = range of compositions for Kīlauea Volcano (Wolfe and Morris, 1996b); data for BC = Big Craters and North Crater flows, HW = Highway and Serrate flows, and KB = Kings Bowl and Wapi flows (for comparison) from Kuntz *et al.* (1992) and unpublished analyses by the authors. Mars data fields summarized by Sautter *et al.* (2015) derived from NASA's Curiosity Rover at Gale Crater, SNC meteorites, and Spirit rover data from Columbia Hills and Gusev Plains.

Differences in composition also reflect intrinsic differences in eruption temperatures and gas composition, both of which could have significant effects on the degree of high-temperature syn-emplacement alteration or the number and types of fumaroles. Lava flows on the northern part of the Great Rift at COTM are chemically evolved from basaltic parents, with possible contamination from assimilation of country rock (Leeman, 1982b). In particular, they have significantly higher alkali metal concentrations (Fig. 6), even when their silica content remains within more primitive ranges. In contrast, the lava flows on Kīlauea and the ERZ are generally basaltic with much more primitive compositions, both with regard to SiO_2_ and total alkali metal content (K_2_O + Na_2_O) (Fig. 6).

Geochemical differences may further be associated with the processes and products of rock alteration and, by inference, they will further modify the availability of nutrients and energy sources for biological activity. Subdued surface weathering likely characterizes present-day Mars such that the rate of rock alteration is currently slow relative to surface processes on Earth. Although analog field targets on Earth are generally younger and therefore will have experienced weathering processes over a shorter time span compared with their martian counterparts, rock alteration on early Mars was possibly more acidic, which would enhance mineral decomposition and the deposition of secondary jarosite (Elwood Madden *et al.*, 2004; Hurowitz *et al.*, 2006). Perhaps more importantly, alteration generated by the interaction of surfaces with hot eruptive volcanic gases or sustained fumarolic activity on either planet will be much faster and produce secondary mineral assemblages distinct from those related to weathering processes.

**FIG. 7.**
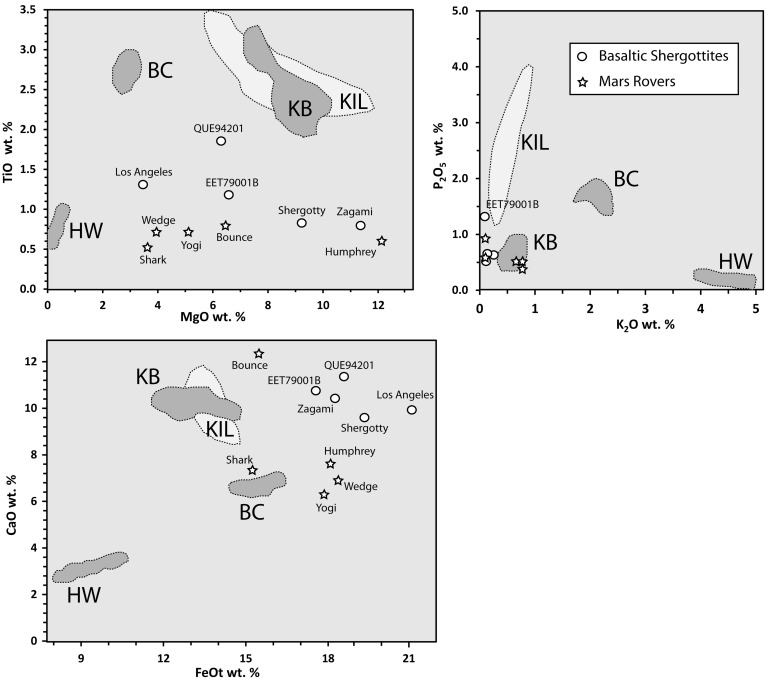
Major oxide analyses of Mars basaltic shergottites and rover targets (Bridges and Warren, 2006) compared to variations in BASALT research target regions and similar lava flows. KIL = Kīlauea; BC = Big Craters and North Crater; HW = Highway and Serrate; KB = Kings Bowl and Wapi shown for comparison to KIL; (data for KIL from Wolfe and Morris, 1996b; data for other fields from unpublished analyses by the authors). BASALT, Biologic Analog Science Associated with Lava Terrains.

Since chemical weathering is likely to be more effective in warm, moist climates such as Hawaii compared with colder, drier conditions in Idaho, differences in types of weathering, as well as the length of exposure time to weathering agents, will have a significant effect on alteration mineralogy. While COTM lavas have been exposed to external weathering agents for a longer period of time compared with Kīlauea, the degree of chemical weathering is significantly lower. Each of the targeted research areas (Figs. 4 and 5) has unique compositional, lithologic, and textural properties associated with primary and secondary processes. Primary differences in geochemical signatures of major elements TiO_2_, K_2_O, P_2_O_5_, MgO, FeO, and CaO (Fig. 7) reflect the various physical properties of targeted regions. The geochemical differences are manifested in different lava flow types, geomorphology, and human accessibility (Fig. 3), which must be investigated via remote sensing (RS) before planning field work. Although significant geologic information is available to field geologists studying Earth, a crewed Mars mission would necessarily rely on RS imagery to determine ground conditions to be expected. The need for this type of preliminary geologic information derived from orbital or robotic imagery, including the types and intensities of rock alteration, cannot be overstated.

**FIG. 8.**
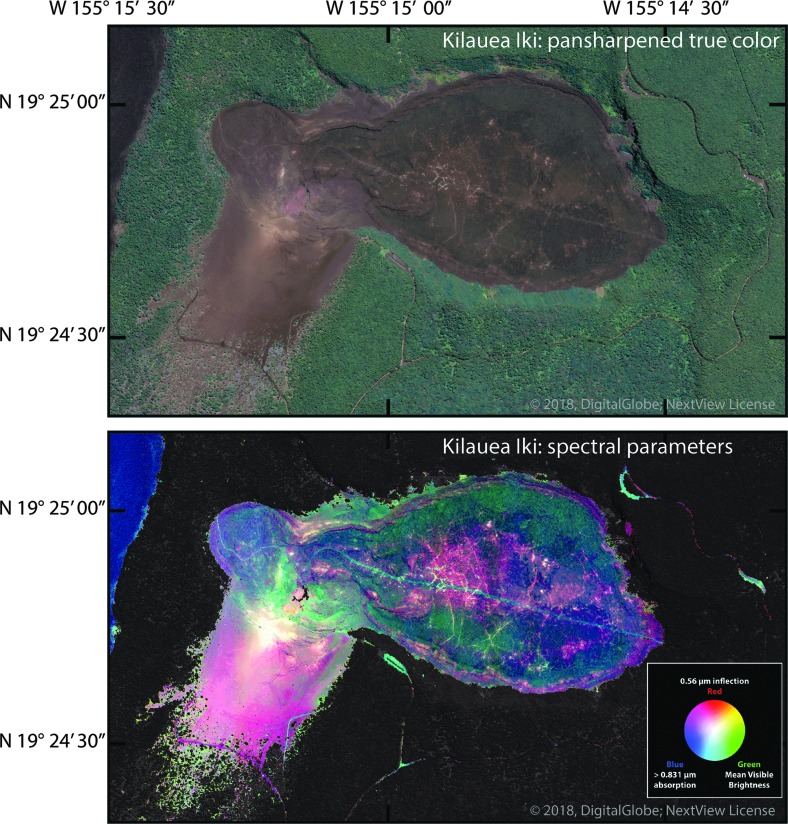
Satellite imagery of the Kīlauea Iki region (RT 4). Upper: true color ∼1 m/pixel pan-sharpened image. Lower: Multispectral false-color image that depicts areas of Fe-oxidation in pink-red, average visible brightness in green, and the presence of iron in mafic minerals in blue.

RS data sets are evaluated here to illustrate the issues that go into mission planning and, especially, daily field excursions. Selection of targeted regions is based on preliminary RS data aimed at specific scientific interest and expected results (Brady *et al.*, 2019). Selection also follows the evaluation of rock alteration determined by RS analysis, that is, the multispectral signatures that provide clues to the causes, mechanisms, and types of rock alteration. An example of RS sensitivity to chemical signatures (*i.e*., mineralogical and alteration properties) is shown in Fig. 8 for the Kīlauea Iki region (RT 4). The two satellite-acquired images illustrate (1) a pan-sharpened ∼1 m/pixel true color scene and (2) a multispectral, false color mineralogical parameter map (Fig. 8, upper and lower, respectively). The false color image enables the distinction of sensitivities to Fe-oxidation (red), average visible brightness, or sulfur deposits (green), and the presence of ferrous iron in mafic minerals (blue). Thus, the detailed evaluation of these types of RS data, a topic to be presented in a forthcoming article, leads to better understanding of how basaltic and similar lava flow types, as well as other volcanic deposits, are affected by liquid water, volatiles, fumaroles, and any other climate condition that will cause changes in the availability of elemental nutrients.

### 3.2. ESRP and the Great Rift

Relatively young and compositionally diverse volcanics comprise a series of tholeiitic basalts and derivatives (hawaiites, latites, and rhyolites) in the upper 1–2 km of the ESRP. The surface morphology of the ESRP, a 400 × 100 km topographic and structural depression, is dominated by basaltic low shields and lava fields that erupted from ∼1–2 m.y. ago to the present; most are younger than ∼400 k.y. The most recent (Holocene) eruptions of both basaltic and evolved lavas on the ESRP (*e.g*., Kuntz *et al.*, 1992; Hughes *et al.*, 1999), including the lava fields of Hells Half Acre, Wapi, Cerro Grande, and Craters of the Moon, have not been covered by light-colored eolian deposits. These lava fields are well exposed in remote imagery (*e.g*., Landsat), and in outcrop, as extensive dark-gray to black patches (Fig. 1, upper). Beneath this series of relatively young volcanics, and in outcrops exposed on the margins of the ESRP, lie much older (∼10 m.y. old) rhyolite ignimbrites and lava flows associated with the Yellowstone hotspot (Armstrong *et al.*, 1975; Pierce and Morgan, 1992; Smith and Braile, 1994), which is currently active ∼200 km east of COTM.

Each basaltic low shield was constructed by a series of low-viscosity, tube-fed basalt lava flows that initially erupted from fissures oriented perpendicular to the WSW-ENE trend of the ESRP. These monogenetic lava flow fields dominate the upper volcanic sedimentary regional ESRP sequence (Greeley, 1982; Leeman, 1982a; Kuntz *et al.*, 1992; Hughes *et al.*, 2002a, 2002b). By contrast, the polygenetic lava flows in the COTM field, which erupted intermittently since ∼15 k.y. ago along the Great Rift, have chemically evolved basalt-like compositions, such as hawaiite and latite (Fig. 6).

The Great Rift is the most predominant of several volcanic rift zones and numerous vent corridors, all of which are aligned perpendicular to ENE-WSW regional Basin-and-Range extension (Kuntz *et al.*, 1992; Hughes *et al.*, 2002b). Most of the low shields on the ESRP were constructed along aligned eruptive vents active during different episodes. While tholeiitic basalts (low shields) dominate the terrain, the COTM system contains numerous steep cones and compositionally diverse lavas that make it unique in the ESRP.

Extensive geologic mapping and petrologic analyses (*e.g*., Kuntz *et al.*, 1992, 2007) show that COTM lava field is the largest, mostly Holocene, basaltic lava field in the conterminous United States covering ∼1600 km^2^ with ∼30 km^3^ of lava flows and other products of volcanism. Several well-known lava flows in the northern part of the Great Rift include Highway flow, North Crater flow, BC flow, Serrate flow, Devil's Orchard flow, and Blue Dragon flow. These flows are depicted schematically as flow lines along with dominant vents (tephra cones) in Fig. 4 (upper), and the lavas exhibit dramatically different colors and surficial morphology in Landsat imagery in Fig. 4 (lower). Significant differences in surface morphology have been assessed during evaluations of these flows as planetary analogues (*e.g*., Hughes *et al.*, 2016; Mallonee *et al.*, 2016; Neish *et al.*, 2017). These flows erupted within a relatively short period of time (∼2100–2200 years BP) during “Eruptive Period A” (Kuntz *et al.*, 1989, 2007) and are mapped as some of the youngest units in the research zone (only Broken Top flow is stratigraphically younger, but still within the short eruptive period).

Preliminary geochemical data from the work of Kuntz *et al.* (1992) and unpublished data by the authors of this study indicate that the Highway and Serrate flows (“HW” in Figs. 6 and 7) are chemically equivalent although they erupted separately. Also, the BC and North Crater flows (“BC” in Figs. 6 and 7) are chemically equivalent to each other, but different from the Highway and Serrate flows. These equivalency associations imply at least two eruptions from each of two magma reservoirs in the northern COTM region. Available geochemical data are insufficient to determine whether there are other equivalency associations within the youngest group of lava flows or if other magma sources are involved.

#### 3.2.1. BC flow (RT 1)

The BC lava flow is a compound hawaiite lava flow that compositionally plots across the boundary between hawaiite (also known as trachybasalt) and mugearite (also known as basaltic trachyandesite) (Fig. 6). Surface morphology, generally a moderate-relief hummocky pāhoehoe (Fig. 3A), varies with distance from the source at BC vents (tephra cones) (Figs. 3B and 4) and with transitions in flow type. Morphological differences are also inherent between individual flow lobes. The morphology of BC flow is characterized by ∼1–4 m relief, with smooth pāhoehoe plateaus, inflated mounds (tumuli), collapse pits, and meter-thick breakout lobes. BC also exhibits broken and rubbly lava crusts, characteristic of slab-pāhoehoe surfaces.

The BC flow was targeted because the composition is similar to true basalt, yet with a higher alkali metal concentration, and lies conveniently between primary ESRP basalt and evolved latitic compositions (Fig. 6). It further represents an analogue for the least-evolved (lowest SiO_2_) alkalic composition of outcrops analyzed by Curiosity Rover at Gale Crater (Sautter *et al.*, 2015). The BC flow was deemed more appropriate than the chemically equivalent North Crater flow because high levels of foot traffic in the North Crater flow area presented a higher potential for contamination through human activities. Actually, the vent area for the BC flow, as opposed to the BC lava flow itself, was selected as a research target for BASALT (RT 1) (Fig. 4) primarily for accessibility concerns, and also because a reconnaissance study of the vent area at BC indicated significant potential for fumarolic alteration along an extension crack located on the west flank. Preliminary sampling and analysis further justified, with NPS permission to venture into otherwise restricted areas, our decision to engage in more detailed analysis.

#### 3.2.2. Highway flow (RT 2)

The Highway flow (RT 2) (Fig. 4) is a thick (up to ∼15 m) chemically evolved latite (“HW” in Fig. 6) flow with a tall and steep flow front and an extremely rugged surface, ∼4–8 m relief, of jagged spires and steep-sided cracks. Generally it can be characterized as an ‘a'ā flow with block and slab-pāhoehoe components (Fig. 3C, D). The rugged high-relief morphology reflects its emplacement as multiple lobes of sluggish, viscous lava from an eruptive vent most likely north of the highway (Fig. 4, upper). The selection of Highway flow as a research target is also based on its highly evolved chemical composition (Fig. 7) that represents similarly evolved martian volcanics such as the highly alkaline (latitic) rocks determined by Curiosity Rover in Gale Crater (Fig. 6). Moreover, the region, although remote and quite rugged, is ideal for access to pristine flow surfaces.

As a research target, regardless of challenging accessibility issues, the Highway flow provides opportunity to evaluate variability in rock textures as well as possible zones of alteration associated with such variances. Notably, the Highway flow has abrupt variations in texture between dense, stony material and vesicular (“frothy”), glassy material. Field investigations and detailed measurements of this bimodal transition (Sandmeyer *et al.*, 2017) indicate that dense layers, which are evenly distributed, are often separated by isolated pods of frothy lava. Foaming of lava to produce frothy textures likely occurred when volatiles either accumulated in the hinges of lava folds or expanded dramatically due to local depressurization in extension cracks. These areas of frothy texture are more conducive to alteration owing to their greater permeability and reactive surface area and potentially provide a more habitable environment.

### 3.3. Kīlauea Volcano and the ERZ

Hawaiian Islands, according to the leading and now widely accepted hypothesis, evolve in stages of volcanic eruption (Clague and Dalrymple, 1987). These stages reflect the development of the magmatic source as the Pacific tectonic plate, on which rides the Hawaiian-Emperor volcanic chain, moves over the mantle-derived Hawaiian hot spot (*e.g*., Wilson, 1963a, 1963b). Four eruptive periods relevant to this idealized model include (1) preshield (submarine eruptions), (2) shield (tholeiitic basalt), (3) postshield (chemically evolved alkalic lavas), and (4) rejuvenated stages (Clague and Dalrymple, 1987; Peterson and Moore, 1987; Moore and Clague, 1992; Clague, 1996). Significant changes in surface morphology occur during these stages, which are largely manifested in the accumulation of primary to evolved lavas over time.

According to the Clague and Dalrymple (1987) model, copious amounts of tholeiitic basalt magma erupt mainly during the shield stage (>95% of the volcano's volume), producing long thin lava flows that lead to an increase in land mass and island size. Kīlauea and Mauna Loa, the closest subaerial volcanoes to the active hot spot, are currently in the shield stage of their development, whereas other volcanoes on the Big Island (Mauna Kea, Hualalai, and Kohala), are transitioning into postshield stages (Clague, 1996; Sherrod *et al.*, 2007).

The shield stage includes caldera collapse, eruptions of caldera-filling lava, and the growth of low shields and cones along magmatic rift zones. Lower volume magma with chemically evolved compositions erupted during the latter part of the shield stage results in steep-sided cinder cones and shorter lava flows. As eruptions diminish, growth stages are ultimately overtaken by loss of land mass due to erosion, landslides, and other geomorphic changes (*e.g*., Stearns, 1946). As the youngest subaerial volcano in Hawaii (and possibly the most active volcano on Earth), Kīlauea exemplifies the shield stage of Hawaiian eruptions with notable activity during the 19th and 20th centuries that has continued unabated into this century. The subaerial sections of Kīlauea Volcano are thus dominated by basalt lavas with only meager transitions to more chemically evolved compositions relative to the compositions of other Hawaiian subaerial volcanoes (*e.g*., Wolfe and Morris, 1996a, 1996b). Over 90% of the volcano is covered in lava flows <1500 years old (Sherrod *et al.*, 2007), and many of these are historical flows.

Eruptions have resulted in significant changes at and near the summit, including pit craters and active lava lakes in the caldera (Peterson, 1967; Holcomb, 1987; Peterson and Moore, 1987; Neal and Lockwood, 2003). Significant activity has also occurred along flanking magmatic rift zones, along the Southwest Rift Zone in 1920, 1971, and 1974, and along the ERZ, which has been nearly continuously active since 1955 (Holcomb, 1987; Sherrod *et al.*, 2007). Regular updates from the US Geological Survey (https://volcanoes.usgs.gov/volcanoes/Kīlauea) indicate that the lower ERZ, which is currently quiet, entered a vigorous eruptive stage in May 2018 that continued for several months along fissures in the area of Leilani Estates. The recent eruption produced a prodigious amount of lava that consumed numerous homes and reached the ocean in several places (https://volcanoes.usgs.gov/volcanoes/kilauea/multimedia_maps.html). Moreover, the summit region deformed considerably due to magma drain-out since our personal observations in 2016 when the Halema‘uma‘u lava lake was full. The resulting rim collapses have apparently compromised one of our research targets (RT 5, Section 3.3.3. Kīlauea caldera).

As with COTM research targets, accessibility and permitting through the HAVO have significantly influenced the selection of research targets at Kīlauea. Research targets were selected to represent (1) different styles of lava eruption and emplacement and (2) different types of alteration related to fumarolic or syn-eruptive gases. These targets include the following: (RT 3) the flanks and proximal lavas of Mauna Ulu, (RT 4) lava lake of Kīlauea Iki, and (RT 5) altered basalts near the SE margin of Kīlauea caldera (Fig. 5).

#### 3.3.1. Mauna Ulu (RT 3)

The ERZ eruptions of Mauna Ulu occurred for ∼2.5 years during 1969–1971 (Swanson *et al.*, 1979) and again for about the same length of time from February 3, 1972, to July 22, 1974 (Decker, 1987; Tilling *et al.*, 1987). Both eruptive episodes produced prodigious amounts of lava that constructed the Mauna Ulu shield in two stages, created a lava lake in the summit, filled nearby pit craters (including the west pit of Makaopuhi Crater), and eventually flowed through an extensive lava tube system to the sea. Details of the 1972–1974 eruptive phase, including the appearance of myriad volcanic features on the surface as well as the relationships to regional ground deformation at Kīlauea, are extensively documented in the work of Tilling *et al.* (1987). According to an assessment of magma transfer by Decker (1987), the heights of Mauna Ulu and nearby Alae, an adjacent low shield, grew significantly during Mauna Ulu activity. These two vents were connected by a subterranean magmatic plumbing system during 1972–1974 resulting in lava lakes in both craters. Moreover, as the 1969–1971 activity waned and the Mauna Ulu lava lake subsided between eruptions, the regional magmatic plumbing system in Kīlauea was manifested in increased summit inflation and a brief eruptive episode (September 1971) in the caldera and Southwest Rift Zone (Duffield *et al.*, 1982).

Basalt lava flows on the flanks of Mauna Ulu have relatively low-relief (∼1–2 m), shelly to dense vesicular pāhoehoe, a morphology that is characteristic of most fresh lavas on Kīlauea (Fig. 3E, F). Meager amounts of weathering have occurred since 1974, and most surfaces remain fairly fresh even though many park visitors have made the popular trek to the crater rim. Steam continues to rise from the summit crater and numerous fumaroles are active near the rim. Fumaroles also appear in a few locations on the lower flanks, notably where lava filled a small pre-existing pit crater. Most of the fumarolic activity (*e.g*., Fig. 3F) is non-sulfurous and appears to be related to recycling of meteoric water rather than primary emission of magmatic gases. Mauna Ulu lavas are considered ideal, in terms of both accessibility and scientific study, for sampling fresh lava and basalt that have been slightly altered by meteoric fumaroles. It also provides good locales to observe and sample lava that has been extensively oxidized by syn-eruptive hot gases, which is evident in exposures of red-orange, highly vesiculated portions of lava exposed in the walls of several flow channels and collapse pits.

#### 3.3.2. Kīlauea Iki (RT 4)

The Kīlauea Iki lava lake was the result of lavas filling the Kīlauea Iki pit crater during the spectacular 1959–1960 summit eruption of Kīlauea Volcano (Richter *et al.*, 1970; Helz and Thornber, 1987). The lake was one of several, including Alae in 1963 and Makaopuhi in 1965, formed by lava infilling into prehistoric pit craters on Kīlauea in the second half of the 20th century; these were soon covered by Mauna Ulu lavas. Lava fountains blasted up to 580 m high during the 1959 episodes at Kīlauea Iki. Lava filled the ∼1.4 × 0.65 km pit (dimensions at lake level) with a 126 m deep lava lake at maximum depth, and spewed reticulite (pumice) and agglutinated spatter, which grew into a 70-m-high tephra cone (Fig. 3G) adjacent to the vent (Neal and Lockwood, 2003). Detailed records and continuous chronological accounting of the Kīlauea Iki lava lake (Richter *et al.*, 1970) demonstrate that magma migration away from the summit region also resulted in summit subsidence, causing the collapse of the molten core of the Halema‘uma‘u lava lake. The lava lake also subsided by ∼15 m (to a new depth of ∼111 m), which left fresh lava covering the lower walls of the pit. These related events (such as others during Hawaiian volcanic stages) signify the importance of Kīlauea's interconnected magmatic plumbing system.

The Kīlauea Iki lava lake remains thermally productive although it subsided and solidified after the eruption. Numerous fumaroles strewn over the surface appear as mounds of inflated, discolored vents that emit hydrothermal gases with temperatures well over ambient conditions. As a research target region, the floor of the lava lake is accessible to a field team, but also is susceptible to tourist visitation, especially along the well-traveled trail across the pit. Targeted regions are thus dependent on maintaining distance from tourist activities and selecting the most remote locations that meet scientific purposes.

#### 3.3.3. Kīlauea caldera (RT 5)

The caldera at Kīlauea's summit, such as other calderas in active shield growth, is a dynamic system that essentially lies directly over the primary magmatic source. Sequential collapse of caldera walls along circumferential normal faults, ascent and withdrawal of magma causing inflation followed by collapse, highly active volcanic fissures aligned along rifts, and circular pit craters (often with lava lakes) are some of the dominant features in Kīlauea caldera.

A growing body of evidence indicates that such features also define the evolution of summit calderas of most basaltic shield volcanoes on Earth and other planetary bodies (Francis and Oppenheimer, 2004). This notion is supported by both Kīlauea and Mauna Loa calderas, which have been described and mapped as structures related to subsidence due to the withdrawal of magma into rift zones on the flanks of the volcano. Walker (1988) suggests that the Hawaiian calderas are shaped by many small events rather than a few great eruptions. The notion of multiple caldera collapses is supported by mapping in the summit region that indicates at least two caldera-forming eruptions at Kīlauea (Neal and Lockwood, 2003).

Complex processes associated with the evolution of Kīlauea caldera are manifested in the 1971 and 1974 eruptive fissure system along the SE margin and the exposure of 18 dikes in the north and west walls of the caldera (Neal and Lockwood, 2003). The selection of the fissure system as a research target region (RT 5) is due to the proximity to Halema‘uma‘u crater, which purportedly lies above or at least near the active magma reservoir (*e.g*., Walker, 1988). This close proximity to the magmatic source most likely provides access to active fumaroles and fumarolic deposits (Fig. 3G) that are derived from magmatic gases rather than circulating meteoric water. Such activity is more likely to produce sulfurous deposits (native sulfur, sulfides, sulfates) with implications for the biological substrates different from those deposited by fumaroles due to heated meteoric water and thereby providing a contrast to the fumaroles found at nearby Mauna Ulu.

## 4. Analogy to Mars

### 4.1. Volcanic settings on Mars

**FIG. 9.**
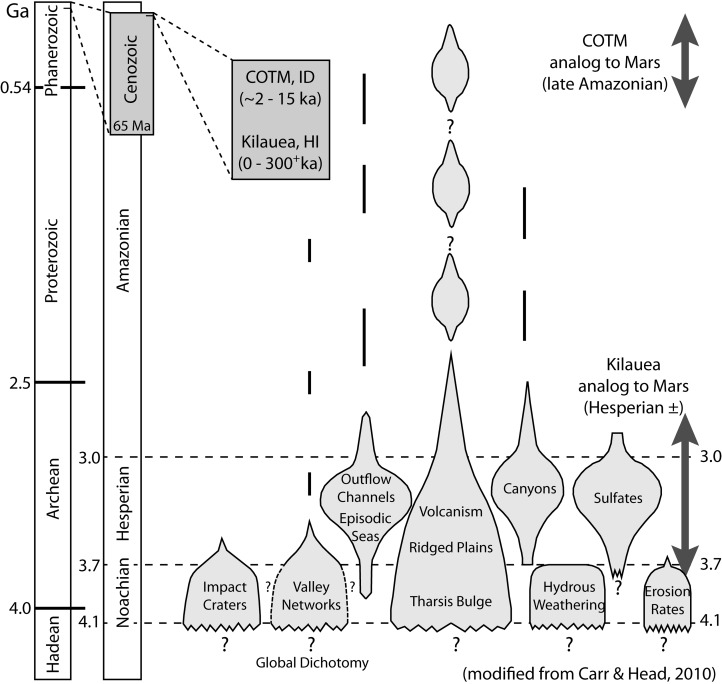
Major geologic events in Mars' history, with relative intensities depicted as variations in width of light-gray shaded regions (Carr and Head, 2010), illustrate significant differences in surface processes that affect climate between early and late Mars. The relatively young ages of research targets at Kīlauea (Sherrod *et al.*, 2007) and COTM (Kuntz *et al.*, 1992) are shown for comparison, with their respective analogs to time spans on Mars shown as dark arrows. Geologic eons (Earth, left and Mars, right) show ages in billions of years (Ga) of the major divisions of geologic time for both planets.

Current knowledge of Mars' geologic history suggests the development of several types of potentially habitable environments, especially in the Noachian period, where abundant meteoric water (including groundwater), evidenced by extensive phyllosilicate deposition and the formation of valley networks (Poulet *et al.*, 2005; Carr and Head, 2010; Ehlmann *et al.*, 2011) was present. Recent work by Ehlmann *et al*. (2011) suggests that the widespread phyllosilicates might be associated with subsurface low-grade metamorphism rather than surface meteoric water. While these environments likely ranged from hot springs (*e.g*., Squyres *et al.*, 2008; Yen *et al.*, 2008; Ruff and Farmer, 2016) to depositional deltas (Carr and Head, 2010), one of the major environmental factors, at least in Mars' early history, was basalt and basalt-related volcanism (Fig. 9). Microorganisms may have grown directly on volcanic rocks, or perhaps at least in materials and fluids produced locally in volcanic terrains without the global presence of meteoric water. Although they are planetary neighbors, Mars and Earth have fundamental chemical and physical differences that impact the nature and style of volcanism on their surface. Cosmochemical bulk composition models suggest that the nebular components that accreted to form Mars were not much different than those that formed Earth (Wänke and Dreibus, 1994; Taylor, 2013).

Differences in oxidation state and subsequent core formation were likely responsible for notable differences in the composition of bulk silicate Mars versus bulk silicate Earth (Taylor, 2013). The most notable difference is the FeO-rich nature of the martian mantle (18.1 wt %) (Taylor, 2013) compared with that of Earth (8.05 wt %) (McDonough and Sun, 1995). This Fe-rich characteristic yields basaltic melts that are enriched in FeO (13–21 wt %) compared with their terrestrial counterparts (∼10–15 wt %); however, the basaltic lavas at COTM have FeO contents at the high end of the terrestrial range (*e.g*., Kuntz *et al.*, 1992; Richardson *et al.*, 2012). Chemical plots (Fig. 7) of SNC basaltic meteorites and rocks analyzed *in situ* by Mars rovers (Bridges and Warren, 2006) also reveal significantly lower TiO_2_ and P_2_O_5_ compared to our basaltic-to-intermediate research targets; whereas MgO, CaO, and K_2_O show no overall differences from the Earth counterparts. In addition to Fe-enrichment, Mars is also thought to be rich in S with mantle and crust concentrations twice that of Earth (King and McLennan, 2010). These differences have important implications for the composition of magmatic products emplaced on the surface (*e.g*., lava flows and magmatic fumaroles).

The cumulative knowledge derived from studies of martian meteorites, orbital spacecraft, and *in situ* lander- and rover-based investigations has shown that the upper crust and much of the surface of Mars are dominated by basaltic materials (McSween *et al.*, 2009; McSween, 2015). Evidence for the existence of more evolved volcanic materials has been identified from orbital spectroscopy investigations (Christensen *et al.*, 2005; Brož *et al.*, 2015b; Rogers and Nekvasil, 2015), analyses from the Mars Exploration Rover Spirit (McSween *et al.*, 2007; Skok *et al.*, 2010), newly discovered martian meteorites (Agee *et al.*, 2013; Santos *et al.*, 2015), and from *in situ* analyses of the Mars Science Laboratory (MSL) in Gale Crater (Sautter *et al.*, 2014; Schmidt *et al.*, 2014; Morris *et al.*, 2016). Analyses by MSL in Gale Crater extend the known martian igneous compositions (Figs. 6 and 7) to much higher alkali and silica contents than previous investigations. In addition, MSL's identification of crystalline tridymite (a high-temperature, low-pressure, SiO_2_ polymorph) suggests that silicic volcanism has occurred on Mars (Morris *et al.*, 2016), further broadening the styles and compositions of volcanism expected for the planet.

Mars' smaller size relative to Earth gives it a larger surface area to volume ratio, which influences the rate at which the planet has cooled, yielding a thick, rigid lithosphere (Schubert *et al.*, 1992; Nimmo and Stevenson, 2001). A thick lithosphere creates a deeper rheological barrier during magma ascent and likely plays an important role in the absence of plate tectonics. Further complicating the process of magma ascent, storage, and emplacement is the lower gravity producing a smaller buoyant force, which drives magma ascent on Earth (Wilson and Head, 1994). A lower buoyant force implies that bodies of magma must have a larger volume to ascend.

**FIG. 10.**
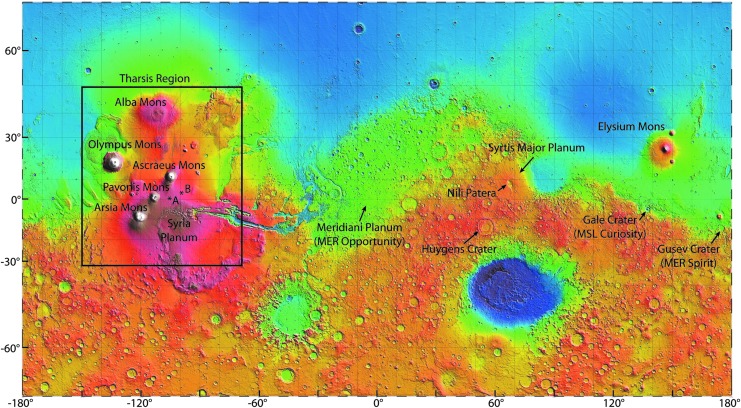
Locations of features on Mars referred to in text. Map base is digitized topography from the Mars Orbiter Laser Altimeter (MOLA) instrument on the Mars Global Surveyor, provided by the NASA Goddard Space Flight Center. Points A and B in the Tharsis region are the locations of volcanic features shown in Fig. 11.

The net result of these physical differences is reflected in the ages of major geologic and climatic events throughout Mars' geologic history (Carr and Head, 2010). Most of the volcanic and surface processes related to weathering, transport, and deposition occurred much earlier (billions of years ago) in the planet's history compared with the young ages (within the last few thousand years) of our terrestrial target regions (Fig. 9). Mars' intense geologic processes early during planetary evolution also resulted in the emplacement of more voluminous lava flows with greater effusion rates from larger dikes tapping deeper magma reservoirs (Wilson and Head, 1994; Greeley *et al.*, 2000). Coupled with the absence of plate tectonics, this allows the construction of massive volcanic edifices such as the great martian shield volcanoes Olympus Mons, Alba Mons, Ascraeus Mons, Pavonis Mons, and Arsia Mons of the Tharsis region (Fig. 10). These prominent landforms reflect a style of volcanism characterized by voluminous eruptions of basaltic lava that form large central shield volcanoes surrounded by many fissures, lava flows, and smaller (low) shields. Although the Tharsis region dominates the volcanology of Mars, other volcanic provinces are significant, including but not limited to Elysium Mons and Nili Patera, which is the summit caldera of the great Syrtis Major Planum volcano (Fig. 10). The dramatic age differences (Fig. 9) and the greater relative intensity of early Mars geologic processes attest to the rationale of evaluating field targets from multiple settings.

**FIG. 11.**
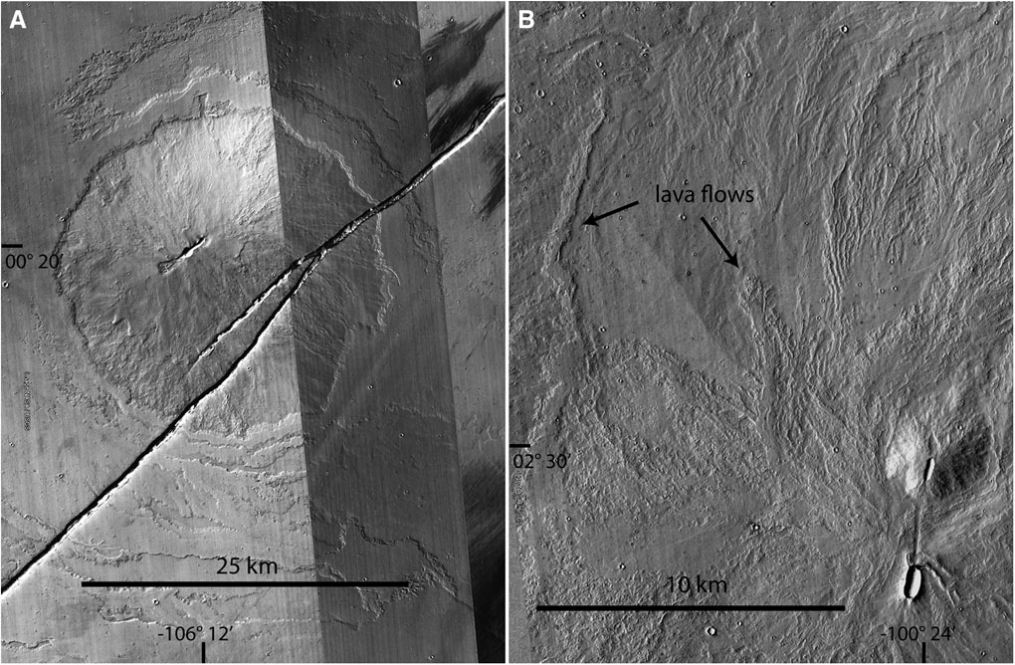
Grayscale CTX composite images of low shields, lava flows, and fissures located east of Pavonis Mons in the Tharsis region (A and B in Fig. 10). **(A)** Illustrates an ∼25-km-diameter low shield, centered at 00°20′ latitude -106°12′ longitude, surrounded by lava flows and incised by a major fissure. **(B)** Illustrates a smaller ∼3 km cone, centered at 02°30′ latitude -100°24′ longitude, with N-S eruptive fissure that produced lavas exposed as flow lobes west and north. Volcanic features in both images are represented in Idaho and Hawaii analog terrains. Mars Reconnaissance Orbiter CTX images obtained from Google Earth. CTX, Context Camera.

Two other prominent basaltic volcanic features of Mars are the Hesperian ridged plains, which were extensive flood basalts that resurfaced nearly 30% of the older Noachian crust (Head *et al.*, 2006), and the less prominent yet ubiquitous (and younger) basaltic plains volcanism (Plescia, 1981; Hauber *et al.*, 2009) located mostly within the Tharsis region. Hesperian flood basalts, while not readily recognized as volcanic features on Mars, are represented by numerous low ridges interpreted by Head *et al.* (2006) as erosional remnants of extensive dikes exposed north and east of Huygens Crater (Fig. 10). Plains-style volcanism, for which the ESRP is a well-exposed, relatively young terrestrial analogue, comprises coalescent low shields and their eruptive fissures, multiple extensive lava flows, and spatter and scoria cones (Greeley and King, 1977; Greeley, 1982). On Mars, basaltic plains-style volcanism has occurred relatively recently in the Tharsis region (Hiesinger *et al.*, 2007), perhaps within the last 100 million years (Hauber *et al.*, 2011), implying the possibility of a present-day dynamic system. Numerous low shields, lava flows and other volcanic features are evident in orbiter imagery (NASA Planetary Data Systems, https://pds.nasa.gov). Examples of relatively small plains-style shields, lava flows, and fissures on the eastern flank of the Tharsis region (Fig. 11), imaged by the Context Camera (CTX) on the Mars Reconnaissance Orbiter (MRO), illustrate similar scale and morphology of martian features to Hawaii and Idaho analogues.

Despite the chemical and physical differences between Mars and Earth, the study of martian meteorites and extensive orbital and *in situ* spaceflight exploration have demonstrated that martian volcanic landforms and compositions are remarkably similar to those found on Earth in intraplate tectonic settings (Greeley and Spudis, 1981; Hauber *et al.*, 2009). These intraplate settings (*e.g*., continental basaltic plains and ocean islands) represent an analogous system for comparison of the volcanic processes occurring on Earth and Mars.

### 4.2. ESRP and the Great Rift

Volcanism on the ESRP provides numerous geomorphologic features considered to be analogues to volcanism recognized on Mars where plains-style volcanism occurred largely in the Tharsis region and locally in the Elysium Mons region (Hauber *et al.*, 2009, 2011). Comparison of low-shield volcanoes in the ESRP and in Syria Planum on Mars using multivariate cluster analysis reveals that (1) plains volcanism is morphologically distinct from other expressions of terrestrial volcanism and (2) the martian low-shield and ESRP volcanoes define this distinct morphological group (Henderson, 2015). This research demonstrates that the martian and terrestrial volcanoes form a morphological spectrum where ESRP volcanoes are smaller and the low shields of Mars are systematically larger, which is to be expected given the physical differences between the planets.

Compositional diversity in volcanic rocks of the ESRP provides the association of geochemistry with geomorphology (Kuntz *et al.*, 1992; Hughes *et al.*, 1999). Low shields and lava fields on the ESRP are typically basaltic, whereas the scoria cones and thick lava flows of the COTM volcanic field have intermediate compositions relatively enriched in SiO_2_, alkalis, FeO, TiO_2_, and P_2_O_5_ compared to the tholeiitic basalt that comprises much of the surrounding regions. These intermediate compositions have similarities to the alkaline volcanic rocks of Gusev Crater and clasts within the martian meteorite NWA 7034 (Usui *et al.*, 2008; Adcock *et al.*, 2018). An important consideration is that the chemical signatures, especially the compositions of exposed rock, may reflect variable amounts of postemplacement alteration. Although the COTM rocks were emplaced between 18,000 and 2000 YBP, their weathering rates are very low and controlled by glass dissolution (Adcock *et al.*, 2018). The young age and arid climate of COTM may indicate only minimal modification by interaction with the environment. Thus, any alteration observed on COTM flows is more likely due to syn-emplacement gas interaction and/or postemplacement fumarolic processes.

#### 4.2.1. BC vent and flow

The source of the BC flow is a vent located at the base of a series of nested scoria cones elongated along the trend of the Great Rift fissure system. This configuration is common for terrestrial basaltic eruptions and the COTM lava field. Scoria cones have been suspected on Mars (Bleacher *et al.*, 2007, 2009), but only recent advances in imaging have allowed detailed examination of these features (Brož *et al.*, 2015a). Differences in shape and form occur between martian and terrestrial cones owing to differences in atmospheric thickness and gravity, but overall there are many similarities, including the close association with lava flows near their base (Hauber *et al.*, 2009; Brož *et al.*, 2015a).

#### 4.2.2. Highway flow

The morphologically and chemically distinct Highway lava flow at COTM provides a unique analogue to less ubiquitous, more evolved Mars volcanic features. The identification of small-scale volcanic edifices with morphologies and spectral properties consistent with the eruption of chemically evolved (*e.g*., Figs. 6 and 7), viscous lavas reveals that Mars is host to localized occurrences of more silicic expressions of volcanism (Christensen *et al.*, 2005; Skok *et al.*, 2010; Brož *et al.*, 2015b; Rogers and Nekvasil, 2015). These flows are exposed in the central caldera of Nili Patera (also known as Syrtis Major) and within the southern highlands at Terra Sirenum, locations where the crust is expected to be thick. Magma ascent through the crust would result in multiple levels of storage consistent with the presence of small volumes of evolved lavas. These processes are analogous to the Highway flow where its location on the northern margin of the ESRP leads to a more complicated path of storage, ascent, and eruption resulting in a small-volume, viscous silicic lava flow (Putirka *et al.*, 2009). On Mars, the detection of hydrated silica deposits by the Spirit rover has been interpreted as evidence for a volcanically driven hydrothermal system (Squyres *et al.*, 2008; Skok *et al.*, 2010) that may have supported biological activity. This discovery suggests the preservation of potentially habitable aqueous environments on the martian surface.

### 4.3. Kīlauea Volcano and the ERZ

The Mars Exploration Rover Spirit conducted an extensive investigation of numerous different volcanic features within Gusev Crater. During its 9-year mission, Spirit encountered potentially primitive olivine-phyric basaltic rocks (Monders *et al.*, 2007; Filiberto *et al.*, 2010), basalts, and trachyandesites enriched in alkalis (McSween *et al.*, 2007, 2009), a basaltic pyroclastic deposit called “Home plate” (Squyres *et al.*, 2007), soils composed of extremely high concentrations of either ferric sulfates or opaline silica (Johnson *et al.*, 2007; Squyres *et al.*, 2008; Yen *et al.*, 2008), and silica sinter deposits (Ruff and Farmer, 2016). These features collectively indicate that Spirit was exploring a region of Mars that once hosted active volcanic hydrothermal springs and fumaroles (Squyres *et al.*, 2008; Yen *et al.*, 2008; Ruff and Farmer, 2016). These discoveries coupled with sulfate-rich mineralogy of the martian surface and the detection of jarosite by Rover Opportunity at Meridiani Planum (Squyres *et al.*, 2004) suggest that alteration on Mars occurs under water-limited low-pH conditions (Hurowitz and McLennan, 2007). Interpretations of these observations have important implications for martian habitability and have stimulated numerous investigations of active volcanic hydrothermal environments as analogues to Mars.

The islands of Hawaii are the most accessible, relevant active volcanic environment for these analog studies, having led to a rich heritage of Mars-centric investigations. Studies focusing on variations in alteration of basaltic tephra (*e.g*., acidic, oxidizing, sulfurous, and ambient) collected from the summit regions of Haleakala and Mauna Kea have shown that these materials are a compelling, although incomplete, spectral analogue to the globally distributed martian dust (Bell *et al.*, 1993; Golden *et al.*, 1993; Morris *et al.*, 2000a; Bishop *et al.*, 2007; Hamilton *et al.*, 2008). Near actively outgassing volcanoes, the interaction of acidic solutions derived from magmatic gases with basalt leads to the formation of thin, visible coatings of amorphous silica on basalt and tephra. This occurs in many places across Hawaii and research has focused on this style of alteration, often termed acid-fog, as a process and spectral analogue for regions on Mars showing evidence for silica or “high-silica phases” (Crisp *et al.*, 1990; Schiffman *et al.*, 2006; Minitti *et al.*, 2007; Chemtob *et al.*, 2010; Seelos *et al.*, 2010; Chemtob and Rossman, 2014). A similar, more pervasive style of alteration occurs in and near fumaroles emitting magmatic gases (*e.g*., H_2_O, H_2_S, SO_2_, CO, CO_2_) and is typified by the presence of amorphous silica, sulfate minerals, Fe-oxides, and native sulfur (Morris *et al.*, 2000b; Bishop *et al.*, 2005; McCanta *et al.*, 2014; Yant *et al.*, 2016).

The primary purpose of many of these analog studies is to constrain the geochemistry and mineralogy of altered materials and examine their spectral properties to identify their unique signatures in RS data sets from Mars. We seek to examine these terrains in a similar manner but with an additional focus on the habitability and biodiversity within extreme environments. In contrast to the abundant research into geothermal hot springs, few studies have focused on the microbial habitability of these active volcanic features primarily because it was assumed that they were too hot to harbor life (Brock, 2012) and the technology required for successful DNA extraction in these environments did not exist (Benson *et al.*, 2011; Wall *et al.*, 2015).

## 5. Conclusions

Volcanic features on Earth, as analog environments for early and present-day Mars, ideally must be associated with specific regions on Mars. Mission designs for crews can take on many forms and involve seemingly endless discussions as to target regions, duration, capabilities, and science objectives. Without the benefit of geologic reconnaissance missions on the surface, the analog locations can only be related to general target regions of Mars, with the notion that more specific targets to investigate will become known in time. While much of Mars' surface is basalt or basalt like, regional alteration of volcanic terrains is likely to have occurred early in the geologic history, whereas long-term alteration since then would be related to cold, dry conditions or the interaction of lavas with ice. The analog environments selected for this work, while not exact conditions of Mars' environment, are notably variable in composition and within environmental settings that enable the types of alteration and biological activity to be assessed, with regard to both modern and ancient Mars.

Similar realms on Mars are most likely to be located near vents, for example, near relict fumaroles or in specific parts of flows or vent deposits that have experienced high-temperature oxidation due to volcanic gas emanations. Secondary deposits related to low-temperature meteoric weathering may not be ubiquitous on Mars, but perhaps present in more localized regions. Thus, the importance of this research, in terms of assessing analog environments for potential biologic activity, is to learn what forms of information are necessary to target Mars missions designed to search for evidence depicting pre-existing or current life forms. Premission investigative techniques, including orbiter RS, geologic mapping, and rover-based information, provide significant clues to enable mission planning.

With continued efforts, the current assessment of research targets in Hawaii and Idaho could be expanded to (1) include additional lava flow types and compositions to fill in textural and chemical gaps; (2) venture into active high-temperature systems appropriate to extremophile biota; (3) investigate more remote areas that might require greater logistical support, but may yield significantly different results by comparison; and (4) attempt to gain access to subsurface features (deeper than a few cm) in lava flows. The latter two efforts could include venturing into lava caves, which might provide additional information regarding mineral deposits as well as natural protection from surface exposure.

Basaltic terrains on Earth host a diverse microbiota. Even just 2 months after an eruption, lava is capable of hosting a diverse microbial assemblage that includes the capacity to oxidize sulfur and iron as sources of energy (Kelly *et al.*, 2014). To understand the habitability of basaltic terrains and the biota they may support, we need to have a clear understanding of how primary geological materials chemically weather to produce secondary products, and the influence of these alteration processes on the availability of nutrients and energy. As there are many different alteration processes, one challenge is to define the major types of alteration sequences that can be expected on Mars by using terrestrial analogues. As we have shown here, the basaltic terrains in Idaho and Hawaii have provided a way to characterize the geology and major alteration types. This work provides the foundation for a better understanding of the habitability of specific types of basaltic terrains.

## Abbreviations Used

BASALT: Biologic Analog Science Associated with Lava Terrains
BC: Big Craters
COTM: Craters of the Moon National Monument and Preserve
ERZ: East Rift Zone
ESRP: eastern Snake River Plain
HAVO: Hawaii Volcanoes National Park
MSL: Mars Science Laboratory
ROIs: regions of interest
RS: remote sensing
RT: research target
